# Performance, Barriers, and Satisfaction of Healthcare Workers Toward Electronic Medical Records in Saudi Arabia: A National Multicenter Study

**DOI:** 10.7759/cureus.21899

**Published:** 2022-02-04

**Authors:** Hanan F Al Otaybi, Rajaa M Al-Raddadi, Farah H Bakhamees

**Affiliations:** 1 Department of Family Medicine, King Abdulaziz Medical City, Jeddah, SAU; 2 King Abdullah International Medical Research Center, Ministry of National Guard-Health Affairs, Jeddah, SAU; 3 Department of Family and Community Medicine, King Abdulaziz University Hospital, Jeddah, SAU; 4 College of Medicine, King Saud Bin Abdulaziz University for Health Sciences, Jeddah, SAU

**Keywords:** barriers, electronic medical record, healthcare workers, performance, satisfaction, saudi arabia

## Abstract

Background

Electronic medical record (EMR) systems are nowadays available internationally, including in Saudi Arabia. Nevertheless, there are still many obstacles to overcome before their effective implementation. This cross-national study aimed to investigate the perceptions and practices of healthcare workers toward implemented EMR systems.

Methods

A cross-sectional study was conducted across selected hospitals in the four cities of Al-Ahsa, Dammam, Medina, and Riyadh in Saudi Arabia. Healthcare workers of all specialties were invited to participate in the study during the six-month study period from August 2019 to February 2020. The questionnaire was submitted online through institutional e-mails.

Results

The study included a total of 2684 healthcare providers. Almost half of the respondents (47.1%) were aged between 35 and 50 years. High experience with computer use was observed among 38.3% of them, while 54.3% attended EMR training activities. The performance scores of EMR’s compared to previous routines had a median of 24 (interquartile range {IQR} = 0-38). The satisfaction scores with EMR’s ranged between 16 and 80 with a median of 53 (IQR = 48-61). Older participants (>50 years), non-Saudis, and those who attended EMR training had statistically significant higher scores of both EMR performance and EMR satisfaction, (p<0.001). Those working in other medical specialties (not major) had statistically significant higher scores of EMR performance alone (p<0.001), while general practitioners (p<0.001) and females (p = 0.001) had statistically significant higher scores of EMR satisfaction alone. EMR systems’ positive impact on quality of care was the highest agreed-upon benefit reported, while the temporary loss of access to patient records if computers crashed or power failed was the highest agreed-upon barrier.

Conclusions

The attitude and satisfaction of healthcare workers in Saudi Arabia towards EMR systems are acceptable particularly among those who are older, non-Saudi, and have attended EMR training. Improved quality of care was the main noted benefit of EMR’s, followed by improved productivity. The temporary loss of access to patient records if computers crashed or power failed, followed by privacy and security concerns, was the major EMR barrier mentioned.

## Introduction

An electronic medical record (EMR) system is defined in literature as an electronic record of health-related information on an individual that can be created, gathered, managed, and consulted by authorized clinicians and staff within one healthcare organization [1]. In the last two decades, advances in information communication technologies prioritized the conduct of EMR systems not only in developed countries but also in several developing countries [2].

Users of EMR systems include administrative staff, medical staff, and even patients. However, the main users of EMR’s are the medical staff of physicians and nurses who use the EMR system to electronically access patients’ health information [3]. Awareness and perception of healthcare providers and especially physicians toward the transition from conventional paper medical records to electronic medical records have been studied extensively [4-8]. The results of these studies can be classified as studies with positive attitudes and views and studies with negative attitudes and views [4-8]. These attitudes and views were shown to be affected by several common expectations, such as the ease of use, availability of useful extra features, costs, need for training, and confidentiality and security concerns [9-13].

EMR systems have been noted in a number of studies to improve the healthcare sector’s workflow through minimizing medical errors, reducing cost and treatment time, improving patient care by creating a better linkage to all healthcare providers, and reducing file storage space, supplies, and workers needed for the filing of physical medical records and paper charts [14-16]. Researchers have also demonstrated that EMR systems contribute to medical error prevention by improved communication, accessible knowledge, access to required information such as drug dosages, timely checks, monitoring assistance, decision-making support, and both rapid tracking of and response to adverse outcomes [17].

Nonetheless, the aforementioned EMR-system-based healthcare quality improvements and financial gain depend on reaching the greatest number of physicians using the system in an effective way [3,17]. However, despite the myriad benefits of the EMR system, its widespread adoption over the world remains low, and there are still many obstacles to overcome before its effective and successful implementation [3]. DesRoches et al. indicated in their survey that only 4% of ambulatory physicians reported having an effective and fully functional EMR system, while 13% reported having a basic system [3]. Several technological impacts and social issues have slowed the pace of implementation or even prevented the widespread plan of EMR implementation. Previous research, especially in the field of medical informatics, has identified some of the barriers to HIS system adoption among physicians [18]. Among the most common reported barriers were the high cost and insufficient return on investment for small practices and safety net providers, underestimation of the organizational capabilities and change management required, failure to redesign the clinical process and workflow to incorporate the electronic systems, concern that systems will become obsolete, lack of skilled resources for implementation and support, and concerns regarding negative unintended consequences of technology [18].

Saudi Arabia has prioritized the development of e-health as well as the transition from paper-based health records to electronic health records [19]. The Saudi government has adopted “a safe quality healthcare system based on patient-centric care guided by standards, enabled by e-health” as its e-health mission [19]. As a result, several Saudi hospitals have adopted EMR systems [19]. However, although being prioritized by the Saudi government, there has been no formal large-scale evaluation of EMR use in Saudi hospitals. The present study aimed to cross-nationally assess the attitudes, practices, and satisfaction of healthcare workers toward the implementation of a single EMR system across several health facilities in Al-Ahsa, Dammam, Medina, and Riyadh in Saudi Arabia. Moreover, it aimed to identify the perceived benefits of EMR’s and the barriers faced in their implementation.

This article was previously presented as a poster in the College of Medicine Third Annual Research Forum at King Saud Bin Abdulaziz University for Health Sciences, Jeddah, Saudi Arabia, on February 18, 2021.

## Materials and methods

After obtaining the institutional review board approval, a cross-sectional study was conducted across a large Saudi government-funded health system in selected health facilities in the four cities of Al-Ahsa, Dammam, Medina, and Riyadh in Saudi Arabia. Healthcare workers of all specialties were invited to participate in the study during the six-month study period from August 2019 to February 2020. All healthcare workers of all specialties, whether medical or non-medical, who were working at the health facilities during the six-month study period from August 2019 to February 2020 were invited to participate in the study. Males and females from all nationalities were invited to participate in the study with no exclusion criteria.

The questionnaire was submitted online through institutional e-mails. The aim of the research and the security confidentiality of the information was explained to prospective participants in order to secure a high response rate. The first section of the questionnaire inquired about healthcare workers’ demographics, such as age, gender, nationality, job title, specialty, self-rating of experience with computer, and history of attending EMR training courses. The second part included multiple-choice closed-ended questions with Likert scale responses assigned with a number range of one to five to indicate the degree of acceptance of the item. This part included axes of system information and terminology, screen design and layout, system capabilities, technical support and service, ease of use, questions for comparisons with previous routines, and the perceived effect of EMR’s on performance. The questionnaire has been previously applied in Saudi Arabia, and its validity and reliability were proved [20]. Over a time span of two weeks, the utilized questionnaire attained a test-retest reliability rate greater than 80% from ten physicians [20]. As for the questionnaire's content validity, it was assessed by six physicians with medical informatics expertise [20]. Lastly, 12 academic family physicians appraised the questionnaire for its clarity, relevance, and structure to test face validity [20]. 

A pilot study for the questionnaire was conducted by collecting the results of a sample in Jeddah, Saudi Arabia [21]. It provided a trial run for the questionnaire, which involved testing the questions’ wording, identifying ambiguous questions, testing techniques used to collect data, and measuring the effectiveness of a standard invitation to respondents. Accordingly, the questionnaire was adapted and modified.

Data were analyzed using the Statistical Package of the Social Sciences (SPSS) version 25 (Armonk, NY: IBM Corp.). Continuous variables were presented as means and standard deviations, while categorical variables were presented as frequencies and percentages. Scores of performance and satisfaction were computed and tested for normality using the Shapiro-Wilk test. Since the data were abnormally distributed, non-parametric statistical tests were applied. The Mann-Whitney test was utilized for two-group comparisons, while the Kruskal-Wallis test was utilized for more than two-group comparisons. P-values < 0.05 were considered statistically significant.

## Results

The study included 2684 healthcare providers from all different specialties and four cities (Table 1). Most of the respondents (76.9%) were from Riyadh. Almost half of the respondents (47.1%) were aged between 35 and 50 years, whereas 40.2% were aged below 35 years. About two-thirds of them (63.3%) were females. Saudi nationals represented 61% of the respondents. Regarding their specialty, 38.1% were nurses and 12.1% were administrators.

**Table 1:** Socio-demographic profile of the respondents (n = 2684) *Not major specialties.

Factors		Frequency (n)	Percentage (%)
Region	Riyadh	2065	76.9
	Medina	263	9.8
	Al-Ahsa	222	8.3
	Dammam	134	5.0
Age (years)	​​​​​​​<35	1040	40.2
	​​​​​​​35-50	1217	47.1
	​​​​​​​>50	327	12.7
Gender	​​​​​​​Female	1699	63.3
	​​​​​​​Male	985	36.7
Nationality	​​​​​​​Saudi	1027	61.0
	​​​​​​​Non-Saudi	657	39.0
Specialty	​​​​​​​Nurse	1023	38.1
	​​​​​​​Administrative	326	12.1
	​​​​​​​Others	242	9.0
	Pharmacist	149	5.6
	Pediatrics	99	3.7
	​​​​​​​Other medical specialists*	94	3.5
	​​​​​​​Obstetrics and gynecology	82	3.1
	​​​​​​​Lab technician	81	3.0
	​​​​​​​Family medicine	70	2.6
	​​​​​​​Intensive care	68	2.5
	​​​​​​​Surgery	67	2.5
	​​​​​​​Cardiology	58	2.2
	​​​​​​​Radiology	58	2.2
	​​​​​​​Emergency medicine	56	2.1
	​​​​​​​Internal medicine	56	2.1
	​​​​​​​Dentistry	40	1.5
	​​​​​​​Physiotherapist	36	1.3
	​​​​​​​Anesthesia	22	0.8
	​​​​​​​Ophthalmology	21	0.8
	​​​​​​​General practitioners	19	0.7
	​​​​​​​Nephrology	17	0.6

More than half (60.1%) of the healthcare providers appraised their skills and experience with computers to be of an average level, whereas high-level skills and experience were reported by only 38.3% of the subjects. A small minority accounting for 1.5% of all the participants reflected that their skills and experience with computers are of a low level. Approximately half of the respondents (54.3%) have attended EMR training, while the remaining 45.7% did not attend any EMR training.

**Figure 1:**
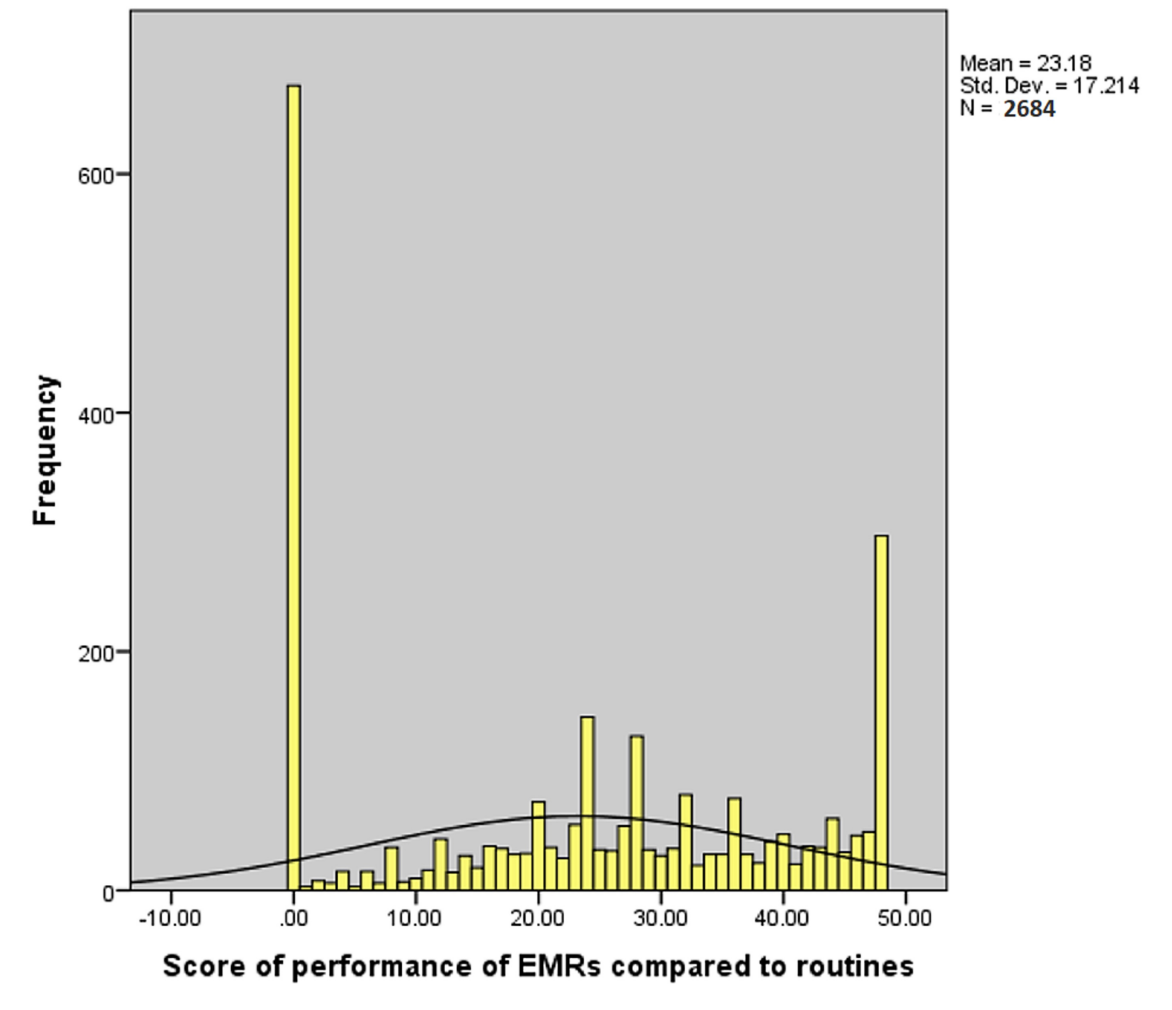
Distribution of healthcare workers’ electronic medical record (EMR) performance scores compared to previous routines.

The healthcare providers` perspectives regarding the comparison between EMR’s to previous routines are summarized in Table 2. More than half of the respondents found that the EMR system is easier than previous routines in seeking out specific information from patient records (61.8%), reviewing the patients' problems (60.4%), obtaining the results from laboratory analyses (60.2%), obtaining the results from x-ray, ultrasound or CT investigations (56%), reviewing currently received medications (55.2%), and entering daily note (53.6%). On the other hand, less than one-third agreed that the EMR system is easier than previous routines in finding patients with certain characteristics (41.6%) and writing prescriptions (31.3%). Overall, the performance score of EMR’s compared to previous routines ranged between 0 and 48 with a median (interquartile range {IQR}) of 24 (0-38) (Figure 1).

**Table 2:** Healthcare workers’ perspectives regarding the change in performance of some tasks when utilizing electronic medical records compared to previous routines.

Healthcare workers’ perspectives	More difficult	No change	Easier	Not applicable
	n (%)	n (%)	n (%)	n (%)
To seek out specific information from patient records	157 (5.8)	146 (5.4)	1658 (61.8)	723 (26.9)
To review the patients problems	127 (4.7)	185 (6.9)	1621 (60.4)	751 (28.0)
To obtain the results from laboratory analyses	84 (3.1)	166 (6.2)	1617 (60.2)	817 (30.4)
To obtain the results from x-ray, ultrasound, or CT investigations	95 (3.5)	185 (6.9)	1504 (56.0)	900 (33.5)
To review currently received medications	173 (6.4)	164 (6.1)	1481 (55.2)	866 (32.3)
To enter daily notes	194 (7.2)	159 (5.9)	1438 (53.6)	893 (33.3)
To find patients with certain characteristics	175 (6.5)	194 (7.2)	1116 (41.6)	1199 (44.7)
To make an appointment	131 (4.9)	155 (5.8)	1013 (37.7)	1385 (51.6)
To order laboratory analyses	97 (3.6)	134 (5.0)	1002 (37.3)	1451 (54.1)
To update diagnoses	89 (3.3)	154 (5.7)	936 (34.9)	1505 (56.1)
To order x-ray, ultrasound, or CT investigations	84 (3.1)	132 (4.9)	898 (33.5)	1570 (58.5)
To write prescriptions	117 (4.4)	110 (4.1)	839 (31.3)	1618 (60.3)

As shown in Table 3, older participants (>50 years) had statistically significant higher scores of EMR performance versus previous routines when compared to those aged <35 years (mean ranks were 1492.15 versus 1263.49), p<0.001. Moreover, non-Saudi nationals had statistically significant higher scores of EMR performance versus previous routines when compared to Saudis (mean ranks were 1469.30 versus 1137.92), p<0.001. Regarding specialty, the highest scores of EMR performance versus previous routines were observed among other medical specialties (mean rank = 1730.69) whereas the lowest scores were observed among laboratory technicians (mean rank = 738.96), p<0.001. Participants who attended EMR training courses had statistically significant higher scores of EMR performance versus previous routines than their counterparts (mean ranks were 1541.35 and 1106.38, respectively), p<0.001.

**Table 3:** Factors affecting healthcare workers’ performance scores in electronic medical records (EMR) compared to previous routines. *Not major specialties. **Kruskal-Wallis test. ***Mann-Whitney test. IQR: interquartile range

Factors		Median	IQR	Mean rank	p-Value
Region	Dammam	25	8-43	1431	0.288**
	Riyadh	24	4-38	1347	
	Al-Ahsa	24	1-36	1316	
	Medina	24	0-39	1282	
Age (years)	<35	23	23-36	1263	<0.001**
	35-50	25	6-39	1365	
	>50	28	15-43	1492	
Gender	Female	24	9-36	1359	0.152***
	Male	24	0-40	1315	
Nationality	Non-Saudi	28	16-40	1469	<0.001***
	Saudi	17	0-35	1138	
Specialty	Other medical specialists*	37	24-45	1731	<0.001**
	Family medicine	39	19-46	1727	
	Surgery	37	21-46	1726	
	Internal medicine	40	20-46	1710	
	​​​​​​​Pediatrics	32	22-45	1670	
	​​​​​​​Anesthesia	37	11-44	1632	
	​​​​​​​Emergency medicine	33	19-43	1625	
	​​​​​​​Obstetrics and gynecology	29	20-44	1606	
	​​​​​​​General practitioner	41	0-45	1604	
	​​​​​​​Dentistry	32	13-43	1555	
	​​​​​​​Cardiology	28	21-41	1547	
	​​​​​​​Ophthalmology	38	0-47	1538	
	​​​​​​​Intensive care	29	19-39	1536	
	​​​​​​​Nephrology	34	8-44	1530	
	​​​​​​​Nurse	28	18-37	1481	
	​​​​​​​Radiology	24	10-36	1330	
	​​​​​​​Physiotherapist	24	0-34	1222	
	​​​​​​​Pharmacist	12	0-28	1002	
	​​​​​​​Others	8	0-25	962	
	​​​​​​​Administrative	0	0-20	799	
	​​​​​​​Lab technician	0	0-16	739	
Experience with computers	​​​​​​​Low	24	0-43	1357	0.137**
	​​​​​​​Average	24	8-38	1366	
	​​​​​​​High	24	0-38	1305	
Attendance of EMR training	Yes	28	19-43	1541	<0.001***
	No	16	0-45	1106	

**Figure 2:**
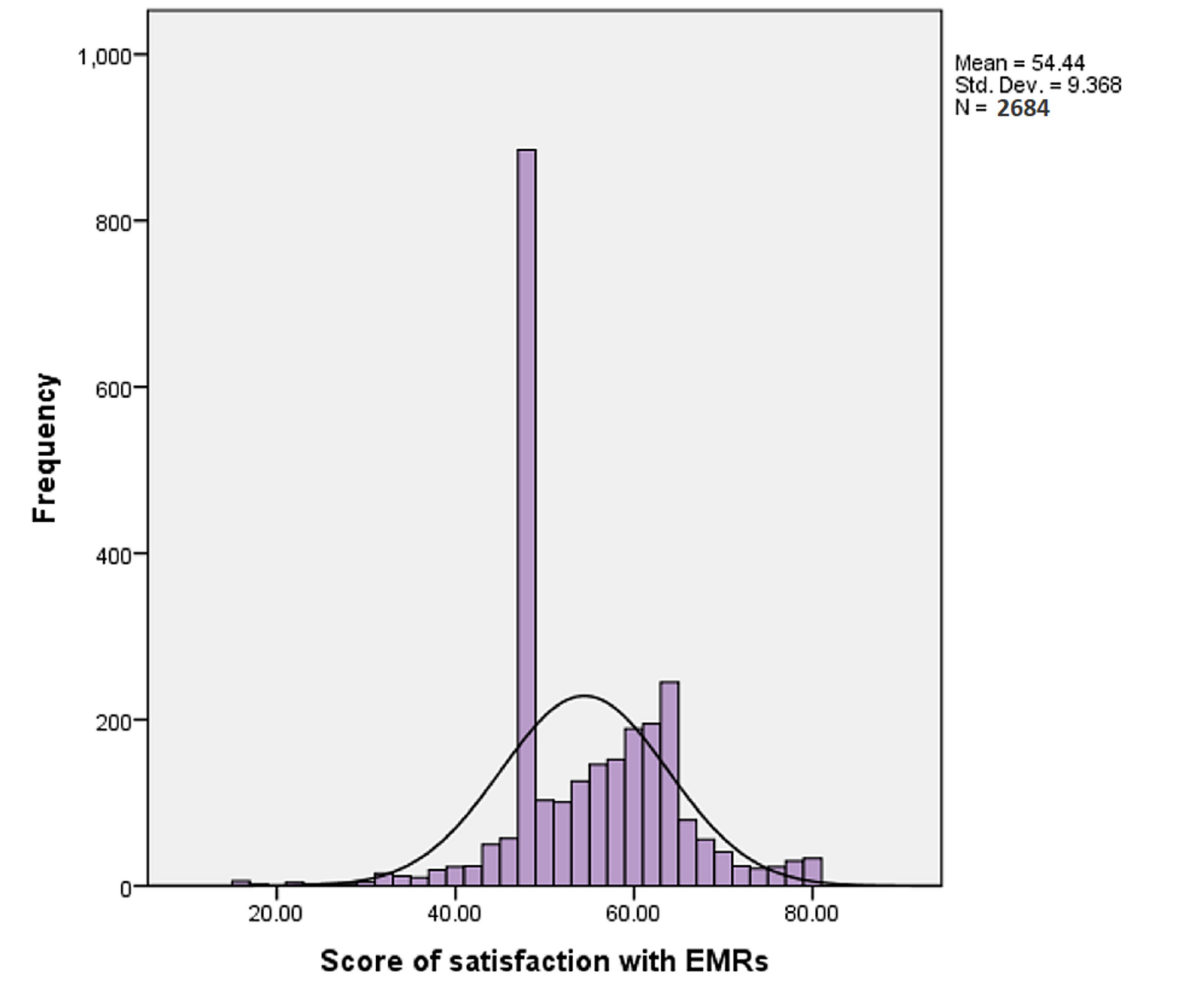
Distribution of healthcare workers’ electronic medical record (EMR) satisfaction scores.

As summarized in Table 4, almost half of the participants (50.3%) agreed that the EMR system provides the precise information they need. However, less than half of the respondents (41%) agreed that templates are well suited to their specialty, and only 45.7% agreed that terminology is related to performed tasks. Moreover, less than half of the healthcare workers (45%) agreed that the EMR system increases their ability to add important content. In regard to design and layout, 51.4% of the participants agreed that the information is clear whereas 48.7% agreed that screen organization is clear. Regarding system capabilities, 35.3% agreed that they rarely experienced difficulty in opening patient file in the EMR system. As for technical support and services, only 37.8% of the healthcare workers agreed that the information technology department provides excellent ongoing technical support and services. Concerning ease of use, 51.7% of the participants agreed that they rarely use the paper-based medical record as an information source in their daily clinical work, and 46.6% agreed that the system is easy to use. Overall, the satisfaction score with EMR’s ranged between 16 and 80 with a median (IQR) of 53 (48-61) (Figure 2).

**Table 4:** Satisfaction of healthcare workers with various electronic medical record (EMR) domains.

		Disagree	Neutral	Agree
		n (%)	n (%)	n (%)
System information and terminology	System provides the precise information I need	107 (4.0)	1226 (45.7)	1351 (50.3)
	Terminology is related to performed tasks	113 (4.2)	1343 (50.1)	1228 (45.7)
	System increases my ability to add important content	145 (5.4)	1332 (49.6)	1207 (45.0)
	Templates are well suited to my specialty	214 (8.0)	1375 (51.2)	1095 (40.8)
Screen design and layout	The information is clear	101 (3.7)	1202 (44.9)	1381 (51.4)
	Screen organization is clear	164 (6.1)	1213 (45.2)	1307 (48.7)
	The output is presented in a useful format	141 (5.3)	1273 (47.4)	1270 (47.3)
	Sequence of screens is clear	165 (6.1)	1258 (46.9)	1261 (47.0)
System capabilities	I rarely experience difficulty in opening patient file in EMR system	367 (13.7)	1369 (51.0)	948 (35.3)
	Unscheduled downtime rarely occurs	354 (13.2)	1476 (55.0)	854 (31.8)
	The system is fast enough	629 (23.5)	1389 (51.8)	666 (24.8)
Technical support and service	IT (information technology) department provides excellent ongoing technical support and services	197 (7.4)	1472 (54.8)	1015 (37.8)
	System reference materials are available	250 (9.3)	1561 (58.2)	873 (32.5)
Ease of use	I rarely use the paper-based medical record as an information source in my daily clinical work	125 (4.7)	1170 (43.6)	1389 (51.7)
	The system is easy to use	162 (6.0)	1243 (46.4)	1279 (47.6)
	The system is user-friendly	182 (6.8)	1254 (46.7)	1248 (46.5)

As demonstrated in Table 5, older healthcare workers (>50 years) had the highest EMR satisfaction scores (mean rank was 1521.98) with statistical significance of p<0.001. Females also had statistically significant higher scores than males (mean ranks were 1379.39 and 1278.87, respectively), p = 0.001. Non-Saudi nationals had statistically significant higher scores than Saudis (mean ranks were 1441.60 and 1282.61, respectively), p<0.001. The highest satisfaction score was reported by general practitioners (mean rank = 1714.39), whereas the lowest score was observed among administrators (mean rank = 1008.29), with statistical significance of p<0.001. Furthermore, healthcare workers who attended EMR training courses expressed statistically significant higher satisfaction scores than their counterparts (mean ranks were 1465.66 versus 1196.25), p<0.001.

**Table 5:** Factors affecting healthcare workers’ satisfaction scores with electronic medical records (EMR). *Not major specialties. **Kruskal-Wallis test. ***Mann-Whitney test. IQR: interquartile range

Factors		Median	IQR	Mean rank	p-Value
Region	Dammam	55	48-62	1404	0.791**
	Riyadh	53	48-61	1341	
	Medina	52	48-62	1336	
	Al-Ahsa	43	48-61	1324	
Age (years)	<35	51	48-61	1315	<0.001**
	35-50	52	48-61	1320	
	>50	57	48-63	1522	
Gender	Female	54	48-62	1379	0.001***
	Male	50	48-61	1279	
Nationality	Non-Saudi	55	48-62	1442	<0.001***
	Saudi	48	48-60	1183	
Specialty	General practitioner	59	48-68	1714	<0.001**
	Family medicine	57	48-63	1623	
	Pediatrics	57	48-64	1546	
	Anesthesia	56	49-63	1527	
	​​​​​​​Obstetrics and gynecology	58	48-62	1509	
	​​​​​​​Emergency medicine	57	48-64	1496	
	​​​​​​​Nurse	56	48-62	1480	
	Surgery	57	48-64	1453	
	​​​​​​​Dentistry	56	48-62	1452	
	Other medical specialists*	56	48-61	1414	
	Cardiology	56	48-63	1444	
	Internal medicine	56	48-63	1388	
	​​​​​​​Radiology	54	48-61	1375	
	Ophthalmology	53	48-59	1267	
	Intensive care	53	47-60	1239	
	​​​​​​​Physiotherapist	48	48-61	1233	
	Others	48	48-58	1148	
	Nephrology	48	48-57	1119	
	Lab technician	48	48-53	1061	
	Pharmacist	48	48-56	1027	
	Administrative	48	48-51	1008	
Experience with computers	​​​​​​​Low	49	48-59	1146	0.242**
	​​​​​​​Average	53	48-61	1349	
	​​​​​​​High	52	48-62	1340	
Attendance of EMR training	Yes	56	48-63	1466	<0.001***
	No	48	48-59	1196	

In regard to the perceived benefits of EMR’s, the highest agreed upon benefit was the positive impact on the quality of care provided (46.1% with a weighted mean on a scale ranging between 1 and 5 equaling to 3.5±0.8) followed by improved productivity (42.1% with a weighted mean of 3.4±0.8). As for the barriers faced with EMR’s, the highest agreed upon barrier was the temporary loss of access to patient records if computer crashes or power fails (41.4% with a weighted mean of 3.4±0.8) followed by privacy and security concerns (33.5% with a weighted mean of 3.3±0.8) (Table 6).

**Table 6:** The perceived benefits and barriers of electronic medical records (EMR) by healthcare workers.

		Disagree	Neutral	Agree	Weighted mean ± SD
		n (%)	n (%)	n (%)	
Benefits	System has a positive impact on quality of care	141 (5.2)	1306 (48.7)	1237 (46.1)	3.5 ± 0.8
	EMR improves my productivity	217 (8.1)	1339 (49.9)	1128 (42.1)	3.4 ± 0.8
	I am able to finish my work much faster than before	283 (10.6)	1334 (49.7)	1067 (39.8)	3.3 ± 0.9
Barriers	Temporary loss of access to patient records if computer crashes or power fails	185 (6.9)	1389 (51.8)	1110 (41.4)	3.4 ± 0.8
	Privacy and security concern	301 (11.3)	1483 (55.3)	900 (33.5)	3.3 ± 0.8
	Lack of ability to achieve a complete paperless system	406 (15.1)	1406 (52.4)	872 (32.5)	3.2 ± 0.8
	Lack of proper doctor-patient communication	375 (14.0)	1470 (54.8)	839 (31.2)	3.2 ± 0.9
	Poor computer skills including typing ability	522 (19.4)	1555 (57.9)	607 (22.6)	3.0 ± 0.8
	EMR increases the risk of making errors	799 (29.8)	1469 (54.7)	416 (15.5)	2.8 ± 0.8

## Discussion

Implementation of health information systems (HIS), such as EMR systems, has been progressing over the past three decades in Saudi Arabia [22,23]. It has been also observed that a number of major Saudi hospitals and healthcare organizations have acclaimed distinguished achievements in EMR implementation in Saudi Arabia, including the Saudi government-funded health system that was the setting of this study [23,24].

In the current study, and in accordance with others, a considerable proportion of the healthcare workers agreed that the EMR system has a positive influence on the quality of care, improves productivity, and enhances the ability of healthcare workers to finish their work considerably faster than before [25,26]. Furthermore, compared to previous routines, more than half of the respondents in this study found that EMR’s are easier in seeking out specific information from patient records, reviewing patients’ problems, obtaining results from laboratory analyses and imaging, reviewing current medications, and entering daily notes. However, they were less satisfied with finding patients with certain characteristics and writing prescriptions. Quite similar findings have been reported previously by others in Saudi Arabia and the United States [20,27,28].

In the present study, the level of experience with computer use had no statistically significant association with healthcare workers' performance and satisfaction with the EMR system. Contrary to this, in a study carried out in Riyadh among physicians and nurses, there was a significant correlation between literacy of computer use and satisfaction with EMR [29]. The difference between both studies could be explained by the inclusion of other categories of healthcare workers, such as including administrative staff, in the present study.

In agreement with others, the attitude of healthcare workers towards EMR’s is encouraging [20,21,30-32]. A considerable proportion of the healthcare workers agreed that the EMR system provides the precise information they need, the templates well suited to their specialty, the terminology related to performed tasks, the clarity of information and screen organization, and the increased ability to add important content. However, almost one-third of the healthcare workers rarely experienced difficulty in opening patient file in the EMR system. While interpreting these findings, we should put in mind that a significant proportion of the participants were neutral in their response as we included different categories of healthcare workers with different interests in EMR’s.

In the current study, preference of the EMR system over the routine paper system was observed more among older participants (>50 years), non-Saudi nationals, those working in other medical specialties (not major), and healthcare workers who attended an EMR training course. In a similar recent study carried out in Jeddah, physicians specialized in internal medicine, obstetrics pediatrics, and family medicine/general practitioners and those who attended EMR training had significantly higher performance scores than their counterparts [21]. In a study carried out in Taif, Saudi Arabia, sex, work department, and familiarity with computer technology were significant predictors for positive attitudes toward EMR [20]. Experience with computers was also a factor significantly associated with the attitude of physicians towards EMR in Eastern Saudi Arabia [30]. In the United States, previous computer experience influenced the positive attitude of healthcare workers towards EMR [5].

Regarding the satisfaction of healthcare workers with EMR’s, half of the healthcare workers in the present survey claimed that the EMR system provides the precise information they need, and a considerable portion reported that templates are well suited to their specialty, terminology is related to performed tasks, and system increases the ability to add important content. In regard to design and layout, almost half of the participants agreed that the information is clear and screen organization is clear. Regarding system capabilities, almost one-third of healthcare workers agreed that they rarely experienced difficulty in opening patients’ files in the EMR system. Concerning technical support and services, more than one-third of the healthcare workers agreed that the information technology department provides excellent ongoing technical support and services. Regarding ease of use, about half of the healthcare workers agreed that they rarely use paper-based medical records as information sources in daily clinical work and that the system is overall easy to use. Overall, the satisfaction with EMR’s was above average. Similarly, in a previous study carried out in Jeddah, most of the healthcare workers agreed that the output of the screen is presented in a useful format, information is clear, screen organization is clear, and sequence of screens is clear [21]. As for system capabilities, half of the subjects in Jeddah’s study agreed that they rarely experienced difficulty in opening a patient’s file in the EMR system and that unscheduled downtime rarely occurs. However, the speed of the system was a concern in Jeddah’s study, and half of the subjects agreed that the information technology department provides excellent ongoing technical support and services and that system reference materials are available. Concerning ease of use, most of them agreed that the system is user-friendly, and they rarely used the paper-based medical record as an information source in their daily clinical work, similar to the findings of this current larger scaled study. Other local and global studies also documented that most physicians were satisfied with EMR services [20,30,33,34]. However, there are others who reported dissatisfaction with the EMR system [35].

In the current study, the commonest reported barriers to the application of EMR’s was the temporary loss of access to patient records if the computer crashed or power failed, followed by privacy and security concerns. The exact same findings have been reported previously in another recent study carried out by our team in Jeddah, Saudi Arabia [21]. Also, in accordance with our findings, Fernández-Alemán et al. raised a concern regarding the confidentiality and security of EMR’s in their study [36]. Additionally, some authors reported the accidental loss of sensitive information from electronic records and even security breaches in healthcare data from both insider and external threats [37,38]. Contrary to this, in a study carried out in Taif, Saudi Arabia, the majority of physicians showed their trust in the confidentiality and security of EMR’s [20].

This study has a few limitations that should be addressed. Inclusion of healthcare workers at one healthcare facility utilizing one EMR system could impact the generalizability of findings over the total population of healthcare workers in Saudi Arabia. Online data collection is considered a limitation; however, it was the only way to reach participants at various distant places in Saudi Arabia. Moreover, the inclusion of all categories of healthcare workers led to a high rate of inconclusive responses to many questions. Finally, as not all participants answered all questions, missing data were particularly noted with the demographic variable of nationality. Despite these limitations, the study has a very large sample size and valued response number, although we used an online questionnaire.

## Conclusions

The attitude of healthcare workers towards the EMR system and their satisfaction with its use are acceptable particularly among older, non-Saudi nationals, and those who attended an EMR training course. The specialty of healthcare workers is an important factor in determining the preference, satisfaction, and utilization of EMR’s over routine paper files. The positive impact on quality of care was the main noted benefit of EMR’s, followed by improved productivity. Based on the healthcare workers' opinions, the temporary loss of access to patient records if computers crashed or power failed, followed by privacy and security concerns, were the most major EMR barriers mentioned.

Based on this study’s results, we recommend the training of healthcare workers in EMR systems, particularly for those with deficient computer experience. Design and layout of the EMR system screen should be improved to be easier and more effective, and the system should be more user-friendly to increase the satisfaction of healthcare providers. Finally, the EMR system should be faster to avoid the loss of healthcare workers’ time and to shorten the waiting time for patients.
