# Correction: Performance, Barriers, and Satisfaction of Healthcare Workers Toward Electronic Medical Records in Saudi Arabia: A National Multicenter Study

**DOI:** 10.7759/cureus.c460

**Published:** 2026-07-06

**Authors:** Hanan F Al Otaybi, Rajaa M Al-Raddadi, Farah H Bakhamees

**Affiliations:** 1 Department of Family Medicine, King Abdulaziz Medical City, Jeddah, SAU; 2 King Abdullah International Medical Research Center, Ministry of National Guard-Health Affairs, Jeddah, SAU; 3 Department of Family and Community Medicine, King Abdulaziz University Hospital, Jeddah, SAU; 4 College of Medicine, King Saud Bin Abdulaziz University for Health Sciences, Jeddah, SAU

This article has been corrected to fix the following typo (in bold) in the Results section: "On the other hand, less than one-third agreed that the EMR system is easier than previous routines in finding patients with certain characteristics (**31.6%**) and writing prescriptions (31.3%)." has been corrected to "On the other hand, less than one-third agreed that the EMR system is easier than previous routines in finding patients with certain characteristics (**41.6%**) and writing prescriptions (31.3%)." This now matches the data displayed in Table 2.

